# Inpatient versus outpatient diagnosis of heart failure across the spectrum of ejection fraction: a population cohort study

**DOI:** 10.1136/heartjnl-2024-324160

**Published:** 2025-01-29

**Authors:** Huan Wang, Chuang Gao, Magalie Guignard-Duff, Christian Cole, Christopher Hall, Resham Baruah, Shikta Das, He Gao, Jil Billy Mamza, Chim C Lang, Ify R Mordi

**Affiliations:** 1 Division of Population Health & Genomics, School of Medicine, University of Dundee, Dundee, UK; 2 Health Informatics Centre, School of Medicine, University of Dundee, Dundee, UK; 3 Medical and Scientific Affairs, BioPharmaceuticals Medical, AstraZeneca UK Limited, London, UK; 4 Division of Cardiovascular Research, School of Medicine, University of Dundee, Dundee, UK; 5 Tuanku Muhriz Royal Chair, National University of Malaysia, Bangi, Malaysia

**Keywords:** Heart failure, Heart Failure, Electronic Health Records

## Abstract

**Background:**

Early heart failure (HF) diagnosis is crucial to ensure that optimal guideline-directed medical therapy (GDMT) is administered to reduce morbidity and mortality. Limited access to echocardiography could lead to a later diagnosis for patients, for example, during an HF hospitalisation (hHF). This study aimed to compare the incidence and outcomes of inpatient versus outpatient diagnosis of HF.

**Methods:**

Electronic health records were linked to echocardiography data between 2015 and 2021 from patients in Tayside, Scotland (population~450 000). Incident HF diagnosis was classified into inpatient or outpatient and stratified by ejection fraction (EF). A non-HF comparator group with normal left ventricular function was also defined. The primary outcome was time to cardiovascular death or hHF within 12 months of diagnosis.

**Results:**

In total, 5223 individuals were identified, 4231 with HF (1115 heart failure with reduced ejection fraction (HFrEF), 666 heart failure with mildly reduced ejection fraction, 1402 heart failure with preserved ejection fraction and 1048 HF with unknown EF) and 992 with non-HF comparators. Of the 4231 HF patients, 2169 (51.3%) were diagnosed as inpatients. The primary outcome was observed in 1193 individuals with HF (28.1%) and 32 (3.2%) non-HF comparators and was significantly more likely to occur in individuals diagnosed as inpatients than outpatients (809 vs 384 events; adjusted HR: 1.62 (1.39–1.89), p<0.001), and this was consistent regardless of EF. For HFrEF patients first diagnosed as inpatients, those discharged on ≥2 GDMT had a reduced incidence of the primary outcome compared with those discharged on <2 GDMT (303 vs 175 events; adjusted HR: 0.72 (0.55–0.94), p=0.016).

**Conclusions:**

Individuals whose first presentation was a HF hospitalisation had a significantly worse outcome than those who were diagnosed in the community. Among hospitalised individuals, higher use of GDMT was associated with improved outcomes. Our results highlight the importance of improving diagnostic pathways to allow for earlier identification and treatment of HF.

**WHAT IS ALREADY KNOWN ON THIS TOPIC**

- Early diagnosis and treatment of heart failure (HF) improve clinical outcomes.
- A HF hospitalisation is a key event in a patient’s clinical course, signalling a period of high clinical risk.
- An increasing number of patients are first diagnosed during a HF hospitalisation rather than in the outpatient setting, and these patients have worse clinical outcomes.

**WHAT THIS STUDY ADDS**

- A first-time diagnosis with HF as an inpatient was associated with an increased risk of death or repeat hospitalisation regardless of left ventricular ejection fraction.
- In those patients who were diagnosed in the inpatient setting, prescribing a greater number of recommended HF therapies at discharge was associated with a reduced likelihood of death or repeated hospitalisation.

**HOW THIS STUDY MIGHT AFFECT RESEARCH, PRACTICE OR POLICY**

- Our study highlights the importance of improving access to key HF diagnostic tests that might allow earlier diagnosis of HF in the community before a hospitalisation.

## Introduction

Despite advances in diagnosis and treatment, heart failure (HF) remains a condition with significant morbidity and mortality.^1 2^ Even in recent large HF outcome trials with well-treated populations, the incidence of cardiovascular death or HF hospitalisation (hHF) over the trial duration was up to 20% at 16 months.^3^ Given the continued poor outcomes, timely diagnosis is key to ensuring that disease-modifying guideline-directed medical therapy (GDMT) can be introduced early to reduce the incidence of adverse events.

A hHF is a key milestone in a patient’s trajectory, signalling an immediate period of high mortality risk and often heralding a long-term decline in HF status.^4 5^ Recent data have shown the importance of not only preventing hHF but also ensuring that if it does occur, the opportunity is taken to maximise GDMT to improve outcomes. This was demonstrated in the recent STRONG-HF trial in which a high-intensity treatment strategy implemented predischarge led to increased GDMT prescriptions and a 34% relative risk reduction in death or HF readmission compared with usual care in individuals hospitalised for HF.^6^ 86% of patients in STRONG-HF had known HF before inclusion into the trial—relatively few individuals had a de novo HF diagnosis.

The majority of studies have examined worsening HF—that is, the impact of hHF in individuals with established HF.^7^,^9^ Fewer studies have evaluated de novo HF diagnosis,^10 11^ and none have compared HF with reduced versus preserved ejection fraction.

The aims of this study were twofold: first, to compare clinical characteristics and outcomes in patients with a new diagnosis of HF stratified by location of diagnosis (inpatient vs outpatient), and second, to compare outcomes in those patients diagnosed for the first time in hospital based on the number of guideline-directed medical therapies at discharge.

## Methods

### Study cohort

Using electronic health records, we evaluated all individuals aged 18 years or older in Tayside, Scotland (current population~450 000), who had either had a first HF hospitalisation or undergone echocardiography for any clinical indication between 1 January 2015 and 30 September 2021. Using the health record data, we excluded individuals with HF hospitalisation prior to January 2015 (dating back to 2002). In Scotland, each person has a unique identifier (the Community Health Index number) that is used throughout all healthcare contacts. Using this identifier, we were able to link demographic, laboratory, echocardiography, hospitalisation and mortality data for follow-up.

For this study, de novo HF was defined as the first instance of any of the three following criteria:

1. A hospitalisation where an International Classification of Diseases (ICD)-10 code for HF was listed as within the first three reasons for admission. The following ICD-10 codes were used: I50, I50.0, I50.1, I50.9, I11.0, I13.0, I13.2, I25.5, I42.0 and I42.9. Where available, these ICD codes were linked to echocardiography to further stratify into heart failure with reduced ejection fraction (HFrEF) (left ventricular ejection fraction (LVEF) ≤40%), heart failure with mildly reduced ejection fraction (HFmrEF) (LVEF 41–49%) and heart failure with preserved ejection fraction (HFpEF) (LVEF≥50%) (inpatient group).
2. In individuals who had never been hospitalised, any echocardiography finding of LVEF<50% (HFrEF or HFmrEF) (outpatient group).
3. In individuals who had never been hospitalised, an echocardiogram with LVEF≥50% and at least two consecutive prescriptions of loop diuretic (outpatient group).

The earliest date of hHF or the earliest date of echocardiography was used as the date of diagnosis of de novo HF. Relevant comorbidities were also ascertained using available laboratory test results, ICD codes and prescribing data as previously described.^12^ GDMT was defined as any prescription of an ACE inhibitor/angiotensin receptor blocker/angiotensin-neprilysin inhibitor (ACEi/ARB/ARNI); beta-blocker; mineralocorticoid receptor antagonist; or sodium glucose cotransporter 2 (SGLT2) inhibitor (SGLT2i), regardless of the prescribed dosage.

A non-HF comparator group was also identified comprising individuals with an echocardiography result demonstrating normal ejection fraction, no record of hHF and no known record of consecutive prescriptions of a loop diuretic during the follow-up period. For this group, the date of echocardiography was used as the start date for follow-up.

### Outcomes

The primary outcome of this study was the time to first incidence of cardiovascular death or hHF. Outcomes were ascertained using data from the Scottish General Register Office. Cardiovascular death was defined as any record within the primary three causes of death recorded under ICD-10 code I00 to I99. Secondary outcomes included hHF alone; cardiovascular death alone; myocardial infarction; stroke; worsening renal function; de novo chronic kidney disease (CKD); and all-cause death. Definitions and code lists used to identify the secondary outcomes are described in online supplemental table S1.

### Statistical analysis

Descriptive statistics were determined for the cohort, including demographic, clinical and laboratory variables. Continuous variables are reported as either mean and SD or median and IQR according to their distribution, with categorical variables reported as count and percentage. Missing baseline covariates were imputed using multiple chained equations with predictive mean matching with 30 imputations.^13^ Associations with clinical outcomes were assessed using Cox regression and Kaplan-Meier analysis. Individuals were followed from their diagnosis of de novo HF for up to 365 days until the first incidence of each study outcome or were right censored at the last date of data availability (ie, ‘2021-12-31’). Multivariable Cox regression analysis included adjustment for relevant clinical variables and recently prescribed medications within 365 days prior to diagnosis of de novo HF. Heterogeneous effects of the diagnostic location, that is, inpatients (late diagnosis) or outpatients (early diagnosis), on the study outcomes across the HF subtypes were tested by including their interaction term in the multivariable Cox model. Stratified analyses were then carried out in subgroups of HF.

To determine the impact of GDMT on discharge from hospital on clinical outcomes, we assessed the time to incidence of repeated hHF or all-cause death in inpatients with HFrEF and HFmrEF who survived up to 28 days from hospital discharge. The association of the number of GDMT at discharge with subsequent clinical outcomes was assessed using multivariable Cox regression, inverse probability of treatment weighting (IPTW) and propensity score matching (PSM). The index date was taken to be 28 days postinitial hHF discharge. Individuals were followed from the index date until the incidence of repeated hHF or all-cause death or censored at the last date of data availability (ie, ‘2021-12-31’). The propensity scores of being prescribed two or more GDMT drug classes (before the index date) were derived from a logistic regression model, including age at initial hHF, sex, deprivation quintile, baseline estimated glomerular filtration rate (eGFR), baseline comorbidities (atrial fibrillation, coronary artery disease, CKD, chronic obstructive pulmonary disease (COPD) and diabetes) and development of worsening renal function during initial hHF. Additional sensitivity analyses were performed: (1) evaluating GDMT at 60 days to allow for a delay in community prescribing; (2) including HFrEF patients only; (3) excluding the small number of elective admissions.

All analysis was performed using R V.4.3.1 within the Trusted Research Environment of the Health Informatics Centre, University of Dundee.

### Patient and public involvement

As an electronic health record study, patients and public were not involved in its conception, design, conduct, analysis or dissemination.

## Results

### Baseline characteristics

**Figure 1:**
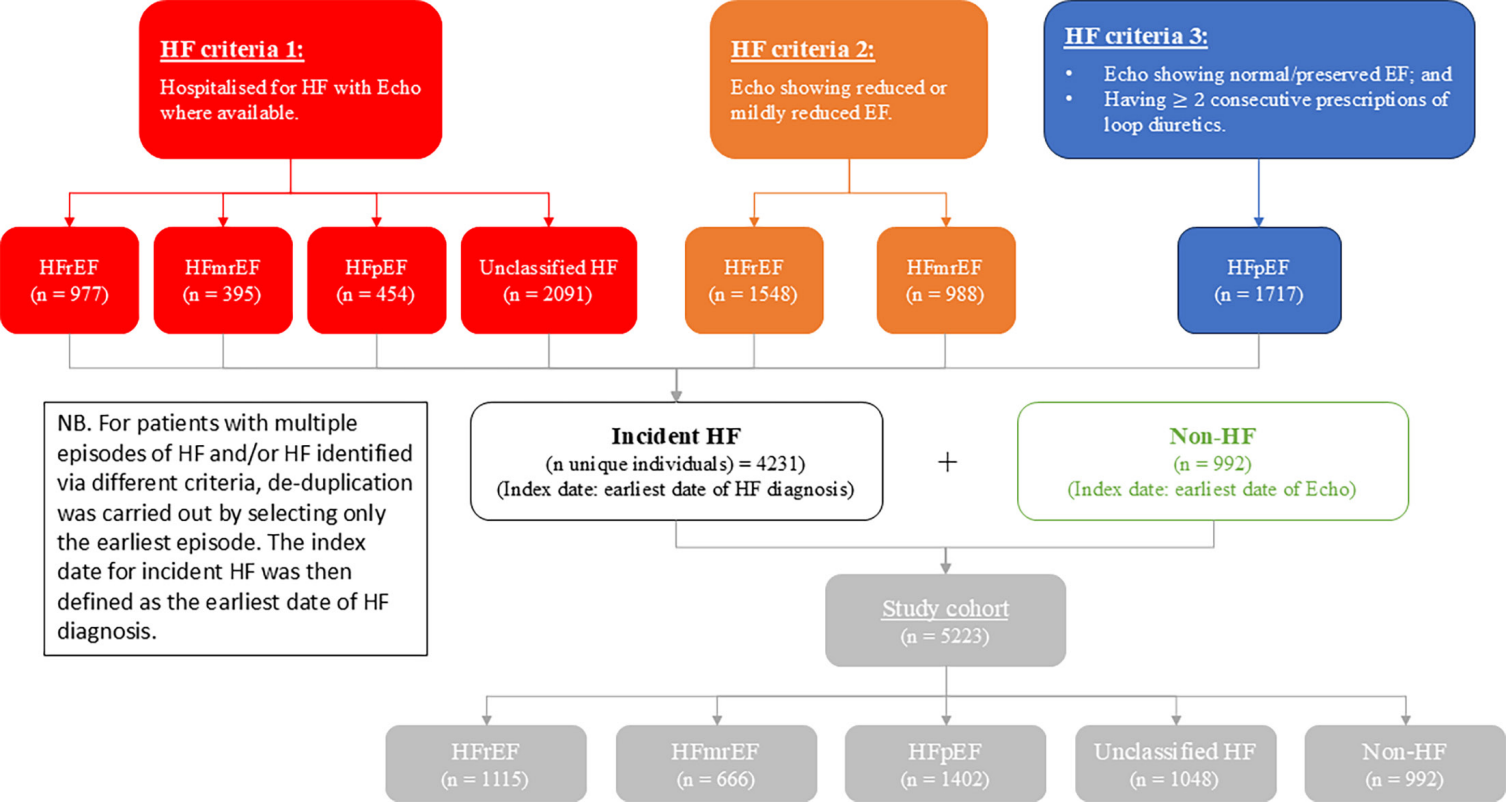
Study flowchart. Cohort derivation. HFmrEF, heart failure with mildly-reduced ejection fraction; HFpEF, heart failure with preserved ejection fraction; HFrEF,heart failure with reduced ejection fraction.

In total, 5223 individuals were included in this study. Cohort derivation is summarised in figure 1. There were 4231 HF patients (1115 HFrEF (26.4% of all HF patients), 666 HFmrEF (15.7%), 1402 HFpEF (33.1%), and 1048 HF with unknown EF (24.8%)) and 992 non-HF comparators. Of the 4231 HF patients, 2169 (51%) were diagnosed as inpatients. Baseline characteristics of the cohort stratified by location of diagnosis are reported in table 1, while online supplemental tables s1 and s2 summarise the baseline data by HF subtype. The mean age of the HF cohort was 72.6 years, and 41.3% were female. Median NT-proBNP was 2147 ng/L. There was a relatively high prevalence of comorbidities, including diabetes (25%), coronary artery disease (35.5%), atrial fibrillation (30.2%), chronic kidney disease (23.9%) and chronic obstructive pulmonary disease (16.5%). There was a relatively low use of GDMT at the time of diagnosis with around a third of patients being prescribed renin-angiotensin-aldosterone inhibitors (36.8%) and beta-blockers (36.4%), and<5% of patients being prescribed MRAs and SGLT2i. Individuals diagnosed as inpatients were more likely to be male with a higher NT-proBNP and a higher prevalence of atrial fibrillation, coronary artery disease, CKD, COPD and diabetes. Two variables had a low amount of missing data, deprivation index (3.8%) and eGFR (2.1%).

**Table 1:** Baseline characteristics at initial heart failure diagnosis

	All HF patients	Inpatients	Outpatients
	(**n=4231**)	(**n=2169**)	(**n=2062**)
Subgroups			
HFrEF	1115 (26.4)	595 (27.4)	520 (25.2)
HFmrEF	666 (15.7)	252 (11.6)	414 (20.1)
HFpEF	1402 (33.1)	274 (12.6)	1128 (54.7)
HF with unknown EF	1048 (24.8)	1048 (48.3)	0 (–)
Age at HF diagnosis—mean (SD)	72.6 (13.3)	72.7 (13.8)	72.5 (12.9)
Female sex	1746 (41.3)	818 (37.7)	928 (45.0)
Scottish Index of Deprivation Quintile			
1 (most deprived)	658 (15.6)	321 (14.8)	337 (16.3)
2	576 (13.6)	311 (14.3)	265 (12.9)
3	723 (17.1)	372 (17.2)	351 (17.0)
4	1398 (33.0)	720 (33.2)	678 (32.9)
5 (least deprived)	717 (16.9)	366 (16.9)	351 (17.0)
Missing (%)	159 (3.8)	79 (3.6)	80 (3.9)
NT-proBNP (ng/L)			
Median (IQR)	2147 (806–4815)	2470 (1235–7069)	2013 (730–4018)
Missing (%)	3996 (94.4)	2097 (96.7)	1899 (92.1)
eGFR (mL/min/1.73 m^2^)			
Mean (SD)	76.7 (24.2)	76.1 (24.4)	77.3 (23.9)
Missing (%)	88 (2.1)	43 (2.0)	45 (2.2)
Comorbidities			
Atrial fibrillation	1276 (30.2)	686 (31.6)	590 (28.6)
Coronary artery disease	1501 (35.5)	1038 (47.9)	463 (22.5)
Chronic kidney disease	814 (19.2)	415 (19.1)	399 (19.4)
Chronic obstructive pulmonary disease	700 (16.5)	351 (16.2)	349 (16.9)
Diabetes mellitus	1058 (25.0)	499 (23.0)	559 (27.1)
Recently prescribed medications*			
ACE inhibitors/ARBs/ARNI	1556 (36.8)	708 (32.6)	848 (41.1)
Beta-blockers	1539 (36.4)	721 (33.2)	818 (39.7)
Mineralocorticoid receptor antagonists	301 (7.1)	132 (6.1)	169 (8.2)
Loop diuretics	1659 (39.2)	568 (26.2)	1091 (52.9)
Aspirin	883 (20.9)	426 (19.6)	457 (22.2)
Statins	819 (19.4)	374 (17.2)	445 (21.6)
Direct oral anticoagulants	706 (16.7)	306 (14.1)	400 (19.4)
Warfarin	243 (5.7)	100 (4.6)	143 (6.9)
SGLT2 inhibitors	63 (1.5)	32 (1.5)	31 (1.5)

* Recently prescribed medications: medications prescribed within 365 days prior to HF diagnosis. Estimates are frequency (%) unless otherwise stated.

ACE, angiotensin-converting enzyme; ARB, angiotensin II receptor blocker; ARNI, angiotensin receptor neprilysin inhibitor; eGFR, estimated glomerular filtration rate; HFmrEF, heart failure with mildly reduced ejection fraction; HFpEF, heart failure with preserved ejection fraction; HFrEF, heart failure with reduced ejection fraction; SGLT2, sodium glucose cotransporter 2.

### Association of diagnostic location with clinical outcomes

**Figure 2:**
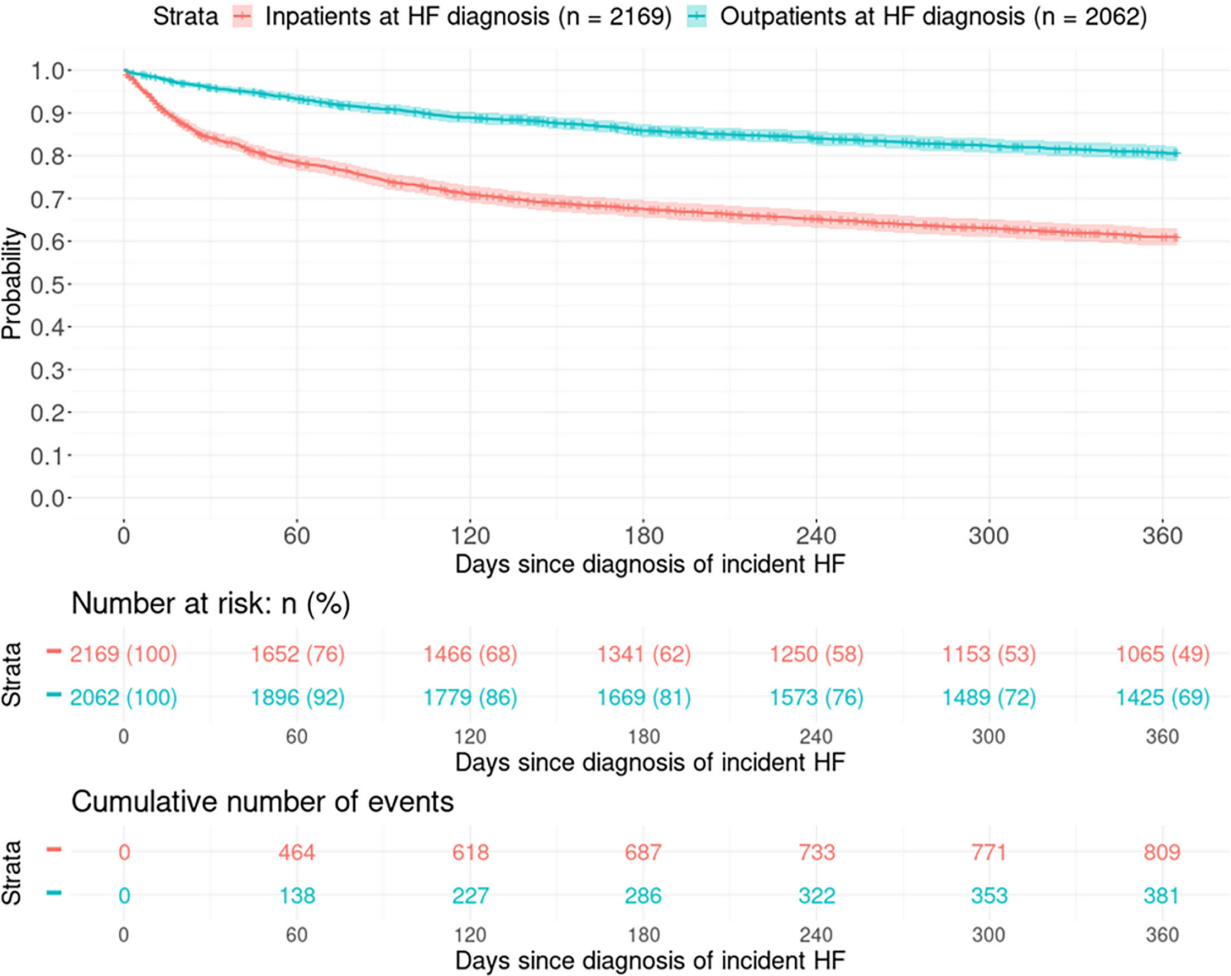
Kaplan-Meier curves of the primary outcome (cardiovascular death or hospitalisation for heart failure in the 365 days postdiagnosis of de novo HF) among inpatients and outpatients. HF, heart failure.

Within the first 12 months following HF diagnosis, the primary outcome of cardiovascular death or HF hospitalisation occurred in 1193 individuals (28% of the total cohort). The incidence of major adverse cardiovascular outcome was higher in individuals diagnosed as inpatients compared with outpatients (online supplemental table S4). For those diagnosed as inpatients, the incidence rate of the primary outcome of cardiovascular death or HF hospitalisation was 58.1 per 100 person-years (95% CI 54.2–62.3) compared with 22.8 per 100 person-years (20.6–25.2) in those diagnosed as outpatients (figure 2). A similar pattern was seen for other cardiovascular outcomes. Incidence rates in HF patients were much higher compared with the non-HF group (online supplemental figure S1). After adjustment for relevant baseline characteristics, patients diagnosed as inpatients had a significantly higher incidence of the primary outcome compared with outpatients (adjusted HR: 1.62 (1.39–1.90), p<0.001). Inpatient diagnosis was associated with increased incidence of the primary outcome regardless of left ventricular ejection fraction (adjusted HR: HFrEF—1.27 (1.04–1.55), p=0.020; HFmrEF—1.52 (1.03–2.23), p=0.034; HFpEF—3.28 (2.37–4.55), p<0.001) (table 2 and online supplemental table S5). Inpatient diagnosis was particularly associated with worse outcome in HFpEF patients compared with HFrEF (interaction p value<0.001). Inpatient diagnosis was associated with a higher incidence of HF hospitalisation in HFpEF (online supplemental figure S2). Results were similar after the exclusion of 204 elective admissions (4.7% of the cohort) (online supplemental table S6).

**Table 2:** Relative risk of the primary outcome (cardiovascular death or hHF) in inpatients compared with outpatients in the 365 days following diagnosis of de novo HF

Cohort	Unadjusted HR (95% CI)	P value	Adjusted HR* (95% CI)	P value
All HF patients	2.39 (2.12 to 2.70)	<0.001*	1.62 (1.39 to 1.90)	<0.001*
HF subgroups†				
HFrEF	1.15 (0.95 to 1.39)	0.138	1.27 (1.04 to 1.55)	0.020*
HFmrEF	1.33 (0.96 to 1.86)	0.088	1.52 (1.03 to 2.23)	0.034*
HFpEF	3.47 (2.59 to 4.65)	<0.001*	3.28 (2.37 to 4.55)	<0.001*

* The HRs were obtained from a multivariable Cox regression adjusted for age at diagnosis of de novo HF, sex, deprivation quintile, baseline eGFR, baseline comorbidities prior to diagnosis of de novo HF (atrial fibrillation, coronary artery disease, chronic kidney disease, chronic obstructive pulmonary disease, diabetes) and medications (ACE inhibitors/ARBs/ARNI, beta-blockers, mineralocorticoid receptor antagonists, loop diuretics, aspirin, statins, direct oral anticoagulants (DOACs) and warfarin) prescribed within 365 days prior to diagnosis of de novo HF.

† No stratified analysis was performed for HF patients with unknown EF as they were all diagnosed as inpatients (see table 1). This subgroup was included in the main analysis of ‘All HF patients’.

ACE, angiotensin converting enzyme; ARB, angiotensin II receptor blocker; ARNI, angiotensin receptor neprilysin inhibitor; eGFR, estimated glomerular filtration rate; HFmrEF, heart failure with mildly-reduced ejection fraction; HFpEF, heart failure with preserved ejection fraction; HFrEF, heart failure with reduced ejection fraction; hHF, HF hospitalisation.

### Association between GDMT and outcomes following hospital discharge in patients with reduced left ventricular ejection fraction

**Figure 3:**
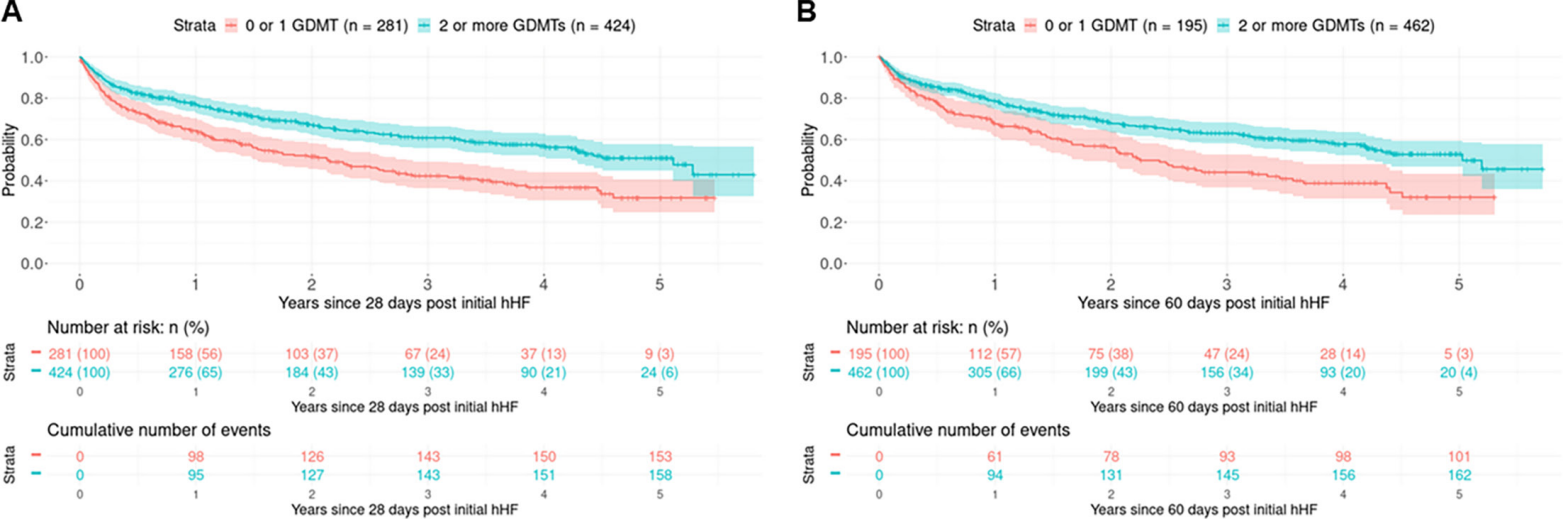
Kaplan-Meier curves of all-cause death or hospitalisation for heart failure (hHF) among heart failure with reduced ejection fraction and heart failure with mildly reduced ejection fraction inpatients from 28 (A) or 60 days (B) postdischarge from initial hHF, stratified by receiving either 0/1 or 2 or more GDMT drug classes (within the first 28 or 60 days posthospital discharge). GDMT, guideline-directed medical therapy.

705 HFrEF/HFmrEF patients diagnosed as inpatients survived to 28 days from hospital discharge (83.2%). Following discharge from a first HF hospitalisation, the majority of these individuals were prescribed either two or three GDMT (more than two medications—424 patients (60.0%) and 0 or 1 medication—281 patients (39.8%)) (table 3). Patients prescribed two or three GDMT on discharge were more likely to be male, with higher NT-proBNP levels, better renal function and fewer comorbidities other than a higher prevalence of coronary artery disease (table 4). Patients discharged taking two or three GDMT had a significantly lower incidence of repeated hHF or all-cause death following discharge than those prescribed suboptimal therapy (adjusted HR compared with those discharged on zero or one GDMT drugs—multivariable Cox regression 0.68 (0.53–0.86), p=0.001; IPTW 0.68 (0.53–0.87), p=0.002; PSM 0.68 (0.53–0.87), p=0.003) (figures3  4). These results were consistent when only HFrEF patients were examined, with individuals prescribed two or three GDMTs at discharge again having reduced incidence of repeated hHF or all-cause death (adjusted HR compared with those discharged on zero or one GDMT drugs—multivariable Cox regression 0.72 (0.55–0.94), p=0.016; IPTW 0.72 (0.54–0.95), p=0.02; PSM 0.72 (0.54–0.96), p=0.024) (online supplemental figure S3). Results were similar in a sensitivity analysis evaluating patients surviving to 60 days.

**Table 3:** Number of GDMT drug classes postdischarge from hospitalisation for heart failure

Number of GDMT drug classes	28-day cohort*	60-day cohort†
0	113 (16.0)	56 (8.5)
1	168 (23.8)	139 (21.2)
2	298 (42.3)	305 (46.4)
3	125 (17.7)	154 (23.4)
4	<5 (<1)	<5 (<1)

Estimates are frequency (%). (Note that due to data sensitivity any numbers <5 are reported as <5, and any percentages <1% are reported as <1%).

* 28-day cohort includes all inpatients with hHF-free survival for at least 28 days postdischarge.

† 60-day cohort includes all inpatients with hHF-free survival for at least 60 days postdischarge.

GDMT, guideline-directed medical therapy; hHF, HF hospitalisation.

**Table 4:** Baseline characteristics of HF patients diagnosed as inpatients used in the 28-day cohort

	Overall	0 or 1 GDMT	2 or more GDMTs
	(**n=705**)	(**n=281**)	(**n=424**)
Subgroups			
HFrEF	478 (67.8)	175 (62.3)	303 (71.5)
HFmrEF	227 (32.2)	106 (37.7)	121 (28.5)
Age at HF diagnosis—mean (SD)	69.6 (13.0)	74.0 (12.9)	66.7 (12.3)
Female sex	209 (29.6)	90 (32.0)	119 (28.1)
Scottish Index of Deprivation Quintile			
1 (most deprived)	118 (16.7)	45 (16.0)	73 (17.2)
2	114 (16.2)	51 (18.1)	63 (14.9)
3	113 (16.0)	41 (14.6)	72 (17.0)
4	222 (31.5)	98 (34.9)	124 (29.2)
5 (least deprived)	113 (16.0)	39 (13.9)	74 (17.5)
Missing	25 (3.5)	7 (2.5)	18 (4.2)
NT-proBNP (ng/L)			
Median (IQR)	4818 (2138–8390)	2858 (1372–7982)	6012 (4668–8390)
Missing (%)	681 (96.6)	269 (95.7)	412 (97.2)
eGFR (mL/min/1.73 m^2^)			
Mean (SD)	79.4 (23.1)	74.0 (25.3)	82.9 (20.9)
Missing (%)	6 (<1)	<5 (<1)	<5 (<1)
Comorbidities			
Atrial fibrillation	187 (26.5)	95 (33.8)	92 (21.7)
Coronary artery disease	417 (59.1)	139 (49.5)	278 (65.6)
Chronic kidney disease	93 (13.2)	62 (22.1)	31 (7.3)
Chronic obstructive pulmonary disease	100 (14.2)	48 (17.1)	52 (12.3)
Diabetes mellitus	149 (21.1)	64 (22.8)	85 (20.0)
Recently prescribed medications**			
ACE inhibitors/ARBs/ARNI	196 (27.8)	88 (31.3)	108 (25.5)
Beta-blockers	169 (24.0)	85 (30.2)	84 (19.8)
Mineralocorticoid receptor antagonists	31 (4.4)	13 (4.6)	18 (4.2)
Loop diuretics	137 (19.4)	75 (26.7)	62 (14.6)
Aspirin	121 (17.2)	62 (22.1)	59 (13.9)
Statins	112 (15.9)	46 (16.4)	66 (15.6)
Direct oral anticoagulants	57 (8.1)	36 (12.8)	21 (5.0)
Warfarin	25 (3.5)	12 (4.3)	13 (3.1)
SGLT2 inhibitors	15 (2.1)	7 (2.5)	8 (1.9)

Due to data sensitivity any numbers <5 are reported as <5, and any percentages <1% are reported as <1%.

* Recently prescribed medications: medications prescribed within 365 days prior to HF diagnosis. Estimates are frequency (%) unless otherwise stated.

ACE, angiotensin-converting enzyme; ARB, angiotensin II receptor blocker; ARNI, angiotensin receptor neprilysin inhibitor; eGFR, estimated glomerular filtration rate; GDMT, guideline-directed medical therapy; HFmrEF, heart failure with mildly reduced ejection fraction; HFpEF, heart failure with preserved ejection fraction; HFrEF, heart failure with reduced ejection fraction; SGLT2, sodium glucose cotransporter 2.

**Figure 4:**
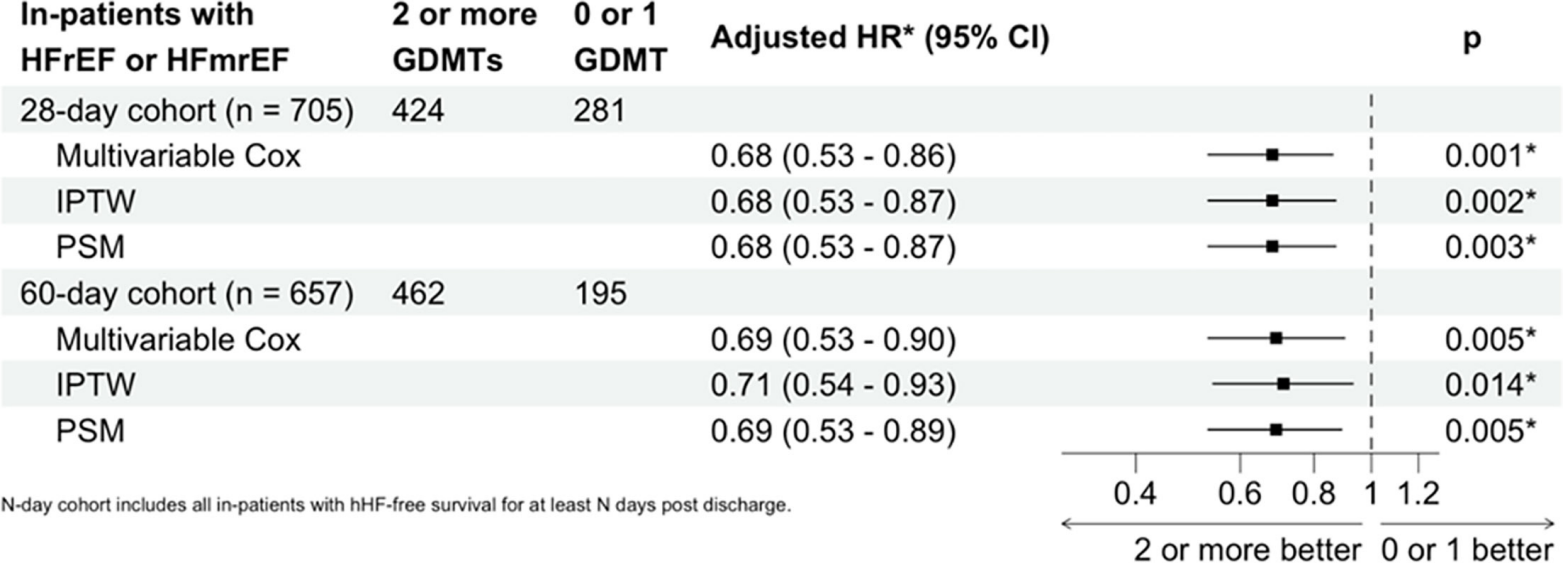
Comparison between 28-day and 60-day GDMT subgroups (among HFrEF and HFmrEF inpatients) on all-cause death or hospitalisation for heart failure. GDMT, guideline-directed medical therapy; HFmrEF, heart failure with mildly-reduced ejection fraction; HFrEF, heart failure with reduced ejection fraction; hHF, HF hospitalisation; IPTW, inverse probability of treatment weighting; PSM, propensity score matching.

## Discussion

In this study, we identified several important findings. First, we found inpatient HF diagnosis was associated with worse outcomes than outpatient regardless of left ventricular ejection fraction. Individuals diagnosed as inpatients were typically older males with a higher prevalence of atrial fibrillation and coronary artery disease. We also found that inpatients with an increased number of GDMT at discharge were associated with improved outcomes. These results highlight the importance of developing strategies to help diagnose HF before a hospitalisation and the need to rapidly optimise HF treatment during discharge for those individuals diagnosed as inpatients.

An HF hospitalisation represents a significant milestone in an HF patient’s clinical care. In a recent survey of HF patients, 1-year mortality following an HF hospitalisation was 23.6%, compared with 6.4% for outpatients with chronic HF.^14^ These results highlight the need to prevent hHF and increase community diagnosis. Our work, in conjunction with other recent studies, demonstrates that the worse outcome after hospitalisation also applies to new HF diagnosis regardless of ejection fraction.^10 15^ Despite the recent development of HFpEF diagnostic scoring systems^16 17^ that integrate clinical features and biomarkers, the diagnosis of HFpEF remains challenging.^18^ These difficulties mean that potential opportunities to prevent hHF by starting SGLT2i and treating comorbidities may be missed by treating clinicians. The particularly poor outcomes associated with inpatient diagnosis of HFpEF may also reflect the lack of evidence-based treatment options in comparison to HFrEF.

Our data support the findings from STRONG-HF that showed the overwhelming benefit of an intensive GDMT optimisation strategy compared with usual care in patients hospitalised with acute decompensated HF.^6^ Only a small proportion of individuals in STRONG-HF did not have a history of HF prior to hospitalisation (15%), and so our study adds to the literature as it suggests that a similar approach to GDMT optimisation could be taken in patients with a new HF diagnosis. Recent randomised trials of SGLT2i^19 20^ and sacubitril/valsartan^21 22^ in patients with worsening HF reinforce this message.

Patients diagnosed as inpatients were more likely to be male with higher NT-proBNP, atrial fibrillation and coronary artery disease. This is similar to previous North American data that suggested that patients first diagnosed with HF were more likely to have a higher prevalence of comorbidities.^23 24^ This probably represents a combination of improved survival from risk factors for HF (eg, myocardial infarction) and a reflection of an increased level of HF severity (development of atrial fibrillation). One particularly striking finding was that patients who were less likely to be prescribed GDMT at discharge were more likely to have a mildly reduced ejection fraction with lower NT-proBNP (although still elevated). This perhaps reflects a ‘grey zone’ in the evidence base around GDMT for this group, and the changes in HF diagnostic criteria over recent years. Nevertheless, the most recent European and American HF guidelines both recommend the use of all four GDMT drug classes in HFmrEF.^25 26^

Our study has some important clinical implications. First, strategies at both clinician and policy level should be put in place to increase opportunities for early HF diagnosis before a hospitalisation occurs. Increased use of natriuretic peptides and improving access to echocardiography are vital to identifying individuals with HF before decompensation. Unfortunately, despite these diagnostic pathways being highlighted in clinical guidelines, they are still underused in routine clinical practice, particularly in primary care settings where many patients may initially present.^27^ As well as improving patient outcomes, earlier diagnosis of HF prior to hospital admission has the potential to provide substantial cost savings.^11^ Although it may not always be feasible to diagnose all HF cases before hospitalisation, previous studies have shown that over a third of patients diagnosed with HF in the hospital had been seen in primary care with HF symptoms in the year preceding their diagnosis—these could theoretically have represented opportunities for diagnosis.^27^ Second, our results highlight that clinicians should use the opportunity of a hHF to optimise GDMT as much as possible prior to discharge. Increased investment in HF specialist services, including clinician and nurse support, may also allow a more intensive monitoring and optimisation strategy akin to that used in STRONG-HF to be put in place in routine clinical care, potentially preventing readmission and death. This approach was highlighted in a recent European Society of Cardiology scientific statement.^28^

A key strength of our study is its comprehensive use of electronic health record data with linkage to prescribing, laboratory tests and importantly echocardiography. This allowed us to evaluate the association of location of diagnosis with clinical outcomes across the ejection fraction spectrum; however, our study does have some limitations. First, as with any observational study, we cannot definitively prove causation. Second, we did not have data on medication dosage, and we were therefore not able to examine the impact of full versus partial optimisation of GDMT. Similarly, we did not have blood pressure data, which might have had an impact on GDMT optimisation. It is possible that we may not have captured all relevant comorbidities as we used ICD coding and prescribing data. NT-proBNP was only introduced into routine clinical practice in Tayside in 2019, so the majority of patients did not have NT-proBNP results available. Nevertheless, it is reassuring that NT-proBNP levels were higher in hospitalised HF patients compared with outpatients, and both were substantially higher than non-HF comparators. We acknowledge that our outpatient diagnostic criteria for HFrEF/HFmrEF could theoretically have included some asymptomatic patients with Stage B HF, although all patients were referred for echocardiography for clinical reasons and therefore are likely to have had some symptoms. Reassuringly, our outpatient HFrEF/HFmrEF patients were closer in clinical characteristics and outcomes to hospitalised patients than to the non-HF comparators. We do note that around 25% of our non-HF comparator group were prescribed a single dose of loop diuretic. Our criteria for a diagnosis of HF required individuals to be prescribed at least two consecutive loop diuretic doses, recognising that in practice patients with breathlessness or oedema may have a trial of loop diuretic pending investigation until an alternative diagnosis is made. It is reassuring though that this group still had a substantially lower NT-proBNP and primary outcome incidence. We have previously shown that this methodology for the identification of HF as applied in our cohort does accurately identify HF patients.^29^ As our study included clinical echos, some echocardiographic parameters, for example, tissue Doppler imaging for the assessment of diastolic function, were not systematically performed. Finally, our data predate the adoption of SGLT2i into widespread clinical use, and so levels of prescribing are low.

## Conclusions

In conclusion, we have shown that, across the ejection fraction spectrum, a first-time diagnosis of HF as inpatient is associated with significantly worse clinical outcomes than diagnosis as an outpatient. We have also shown that better optimisation of GDMT prior to discharge was associated with improved outcome in patients with de novo diagnosis of HFrEF or HFmrEF. Our study highlights the need for strategies to allow earlier diagnosis of HF and intensification of GDMT following an HF hospitalisation.

## Supplementary material

10.1136/heartjnl-2024-324160 online supplemental file 1

10.1136/heartjnl-2024-324160 online supplemental file 2

## Data Availability

Data are available upon reasonable request.
