# Supplementary material for: Inpatient versus outpatient diagnosis of heart failure across the spectrum of ejection fraction: a population cohort study
Source: Heart. 2025 Jan 29;111(11):e324160. doi: 10.1136/heartjnl-2024-324160 (PMC12171480; doi:10.1136/heartjnl-2024-324160)
Supplement: online supplemental file 2 [file heartjnl-111-11-s002.pdf]

Importance of Early Diagnosis and Treatment of Heart Failure across the Spectrum of Ejection Fraction – A Population Cohort Study

Huan Wang[1], Chuang Gao[2], Magalie Guignard-Duff[2], Christian Cole[1,2], Christopher Hall[2], Resham Baruah[3], He Gao[3], Shikta Das[3], Jil Billy Mamza[3], Chim C Lang[4], Ify R Mordi[4]

SUPPLEMENTARY DATA

Supplementary Table S1. Definition and Codelists for Study Outcomes.

Supplementary Table S2. Baseline Characteristics of the Study Cohort.

Supplementary Table S3. Baseline Characteristics of HFrEF and HFmrEF Patients Diagnosed as In-patients vs. Out-patients and Compared to Non-HF Comparators.

Supplementary Table S4. Incidence Rates per 100 Person-Years.

Supplementary Table S5. Full Table for the Primary Outcome with All HF Patients.

Supplementary Table S6. Relative risk of the primary outcome (CV death or hHF) in in-patients compared to out-patients in the 365 days following diagnosis of de novo HF after exclusion of 204 patients with elective hospitalisation as their first event.

Supplementary Figure S1. Kaplan-Meier Plot of the Primary Outcome in In-patient HFrEF/HFmrEF patients vs. Out-patient HFrEF/HFmrEF and non-HF Comparators.

Supplementary Figure S2. Comparison Between In-patients and Out-patients on Secondary Outcomes in the 365 Days post Initial HF Diagnosis.

Supplementary Figure S3. Comparison between 28-day or 60-day GDMT subgroups (among individuals with initial hospitalisation for HF with reduced ejection fraction only) on all-cause death and repeated hHF.

Supplementary Table S1. Definition and Codelists for Study Outcomes.

Study outcome	Definition and codelist
Hospitalisation for HF (hHF)	I50, I110, I130, I132, I255, I420, I429
Cardiovascular (CV) death	I00-I99
Hospitalisation for MI	I21, I22, I252, I256
Hospitalisation for stroke	I60, I61, I62, I63, G45
Worsening renal function (WRF)	**Composite (earliest) of the following:**<br>• A sustained decline in the eGFR of 40% or greater (two consecutive eGFRs below 60% of the baseline level);<br>• Having an eGFR < 15 mL/min/1.73m^2;<br>• Initiated sustained dialysis (appeared on Scottish Renal Registry);<br>• Received kidney transplantation (OPCS code: L74, M012-015, M018, M019, M084, M023, M17, X40-43);<br>• Achieved end-stage kidney disease (ICD-10: E853, N165, T824, T861, Y602, Y612, Y622, Y841, Z490-492, Z940, Z992, N180, N185, Q601).
De novo chronic kidney disease (CKD)	**Composite (earliest) of the following:**<br>• According to the KDIGO definition, the presence of two eGFR records below 60 mL/min/1.73m^2 separated by more than 90 days will be identified as CKD, where eGFR will be calculated using the CKD-EPI Creatinine Equation using standardised serum creatinine level;<br>• ICD-10: N18, N19, I12, I131, I132, E102, E112, E132, Z49, Z992;<br>• Initiated sustained dialysis (appeared on Scottish Renal Registry)

Supplementary Table S2. Baseline Characteristics of the Study Cohort. (Any percentages <1% were reported as <1%.)

	**Overall** (n = 5223)	**HFrEF** (n = 1115)	**HFmrEF** (n = 666)	**HFpEF** (n = 1402)	**HF with unknown EF** (n = 1048)	**Non-HF comparator** (n = 992)
**Age at HF diagnosis** – mean (SD)	72.4 (13.3)	70.9 (13.5)	71.6 (12.7)	72.7 (12.9)	74.9 (13.9)	71.3 (12.9)
**Female sex**	2300 (44.0%)	366 (32.8%)	216 (32.4%)	734 (52.4%)	430 (41.0%)	554 (55.8%)
**Scottish Index of Deprivation Quintile**						
1 (Most deprived)	801 (15.3%)	187 (16.8%)	110 (16.5%)	223 (15.9%)	138 (13.2%)	143 (14.4%)
2	720 (13.8%)	156 (14.0%)	98 (14.7%)	185 (13.2%)	137 (13.1%)	144 (14.5%)
3	906 (17.3%)	189 (17.0%)	102 (15.3%)	236 (16.8%)	196 (18.7%)	183 (18.4%)
4	1735 (33.2%)	361 (32.4%)	221 (33.2%)	450 (32.1%)	366 (34.9%)	337 (34.0%)
5 (Least deprived)	873 (16.7%)	180 (16.1%)	112 (16.8%)	253 (18.0%)	172 (16.4%)	156 (15.7%)
Missing	188 (3.6%)	42 (3.8%)	23 (3.5%)	55 (3.9%)	39 (3.7%)	29 (2.9%)
**NT-proBNP (ng/L)**						
Median [IQR]	1880 [557, 4379]	4263 [2075, 8878]	1978 [1211, 2903]	1046 [244, 2720]	2265 [1386, 6206]	463 [130, 1109]

Missing (%)	4946 (94.7%)	1039 (93.2%)	638 (95.8%)	1306 (93.2%)	1013 (96.7%)	950 (95.8%)
**eGFR (mL/min/1.73m^2)**						
Mean (SD)	77.8 (24.1)	75.5 (24.2)	79.6 (22.3)	78.3 (24.0)	73.9 (25.1)	82.8 (23.1)
Missing (%)	111 (2.1%)	18 (1.6%)	12 (1.8%)	26 (1.9%)	32 (3.1%)	23 (2.3%)
**Comorbidities**						
Atrial fibrillation	1480 (28.3%)	341 (30.6%)	181 (27.2%)	401 (28.6%)	353 (33.7%)	204 (20.6%)
Coronary artery disease	1678 (32.1%)	444 (39.8%)	328 (49.2%)	272 (19.4%)	457 (43.6%)	177 (17.8%)
Chronic kidney disease	930 (17.8%)	195 (17.5%)	106 (15.9%)	254 (18.1%)	223 (21.3%)	152 (15.3%)
Chronic obstructive pulmonary disease	869 (16.6%)	154 (13.8%)	92 (13.8%)	276 (19.7%)	178 (17.0%)	169 (17.0%)
Diabetes mellitus	1285 (24.6%)	271 (24.3%)	161 (24.2%)	387 (27.6%)	239 (22.8%)	227 (22.9%)
**Recently prescribed medications**						
ACE inhibitors/ARBs/ARNI	1898 (36.3%)	381 (34.2%)	239 (35.9%)	569 (40.6%)	367 (35.0%)	342 (34.5%)
Beta blockers	1871 (35.8%)	345 (30.9%)	248 (37.2%)	548 (39.1%)	398 (38.0%)	332 (33.5%)
Mineralocorticoid receptor antagonists	351 (6.7%)	69 (6.2%)	42 (6.3%)	111 (7.9%)	79 (7.5%)	50 (5.0%)

Loop diuretics	1903 (36.4%)	326 (29.2%)	195 (29.3%)	811 (57.8%)	327 (31.2%)	244 (24.6%)
Aspirin	1061 (20.3%)	231 (20.7%)	151 (22.7%)	276 (19.7%)	225 (21.5%)	178 (17.9%)
Statins	1021 (19.5%)	210 (18.8%)	123 (18.5%)	298 (21.3%)	188 (17.9%)	202 (20.4%)
Direct oral anticoagulants	867 (16.6%)	154 (13.8%)	109 (16.4%)	267 (19.0%)	176 (16.8%)	161 (16.2%)
Warfarin	275 (5.3%)	54 (4.8%)	43 (6.5%)	98 (7.0%)	48 (4.6%)	32 (3.2%)
SGLT2 inhibitors	76 (1.5%)	16 (1.4%)	22 (3.3%)	13 (<1%)	12 (1.1%)	13 (1.3%)

Estimates are frequency (%) unless otherwise stated.

HFrEF – heart failure with reduced ejection fraction; HFmrEF – heart failure with mildly-reduced ejection fraction; HFpEF – heart failure with preserved ejection fraction; eGFR – estimated glomerular filtration rate; ACE – angiotensin converting enzyme; ARB – angiotensin II receptor blocker; ARNI – angiotensin receptor neprilysin inhibitor; SGLT2 – sodium glucose cotransporter 2

Supplementary Table S3. Baseline Characteristics of HFrEF and HFmrEF Patients Diagnosed as In-patients vs. Out-patients and Compared to Non-HF Comparators.

	**Non-HF** (n = 992)	**Non-hospitalised HFrEF / HFmrEF** (n = 934)	**Hospitalised HFrEF / HFmrEF** (n = 847)
**Subgroups**			
HFrEF	--	520 (55.7%)	595 (70.2%)
HFmrEF	--	414 (44.3%)	252 (29.8%)
**Age at HF diagnosis** – mean (SD)	71.3 (12.9)	72.1 (13.0)	70.2 (13.3)
**Female sex**	554 (55.8%)	312 (33.4%)	270 (31.9%)
**NT-proBNP (ng/L)**			
Median [IQR]	463 [130, 1108]	2814 [1541, 6693]	4788 [1682, 8766]
Missing (%)	950 (95.8%)	860 (92.1%)	817 (96.5%)
**eGFR (mL/min/1.73m^2)**			
Mean (SD)	82.8 (23.1)	75.8 (23.3)	78.4 (23.9)
Missing (%)	23 (2.3%)	23 (2.5%)	7 (0.8%)
**Comorbidities**			
Atrial fibrillation	204 (20.6%)	294 (31.5%)	228 (26.9%)
Coronary artery disease	177 (17.8%)	295 (31.6%)	477 (56.3%)
Chronic kidney disease	152 (15.3%)	174 (18.6%)	127 (15.0%)
Chronic obstructive pulmonary disease	169 (17.0%)	124 (13.3%)	122 (14.4%)

Diabetes mellitus	227 (22.9%)	241 (25.8%)	191 (22.6%)
**Recently prescribed medications***			
ACE inhibitors/ARBs/ARNI	342 (34.5%)	378 (40.5%)	242 (28.6%)
Beta blockers	332 (33.5%)	381 (40.8%)	212 (25.0%)
Mineralocorticoid receptor antagonists	50 (5.0%)	74 (7.9%)	37 (4.4%)
Loop diuretics	244 (24.6%)	346 (37.0%)	175 (20.7%)
Aspirin	178 (17.9%)	232 (24.8%)	150 (17.7%)
Statins	202 (20.4%)	192 (20.8%)	141 (16.6%)
Direct oral anticoagulants	161 (16.2%)	194 (20.8%)	69 (8.1%)
Warfarin	32 (3.2%)	65 (7.0%)	32 (3.8%)
SGLT2 inhibitors	13 (1.3%)	20 (2.1%)	18 (2.1%)

Supplementary Table S4. Incidence Rates per 100 Person-Years.

	In-patients (n = 2169)			Out-patients (n = 2062)			Non-HF comparator (n = 992)		
**Endpoints**	**Events**	**Person-years**	**Incidence rate (95% CI)**	**Events**	**Person-years**	**Incidence rate (95% CI)**	**Events**	**Person-years**	**Incidence rate (95% CI)**
Primary outcome*	809	1392.21	58.1 (54.2 - 62.3)	384	1684.37	22.8 (20.6 - 25.2)	32	902.34	3.5 (2.4 - 5)
Hospitalisation for heart failure	537	1392.21	38.6 (35.4 - 42.0)	295	1684.37	17.5 (15.6 - 19.6)	0	902.34	(--)
Cardiovascular death	391	1683.76	23.2 (21.0 - 25.6)	142	1841.51	7.7 (6.5 - 9.1)	32	902.34	3.5 (2.4 - 5)
Myocardial infarction	569	1219.33	46.7 (42.9 - 50.7)	186	1716.82	10.8 (9.3 - 12.5)	29	881.62	3.3 (2.2 - 4.7)
Stroke	79	1637.65	4.8 (3.8 - 6.0)	51	1815.33	2.8 (2.1 - 3.7)	30	884.47	3.4 (2.3 - 4.8)
Worsening renal function	238	1594.70	14.9 (13.1 - 16.9)	211	1752.46	12 (10.5 - 13.8)	70	871.25	8 (6.3 - 10.2)
De novo chronic kidney disease	142	1382.84	10.3 (8.6 - 12.1)	116	1462.00	7.9 (6.6 - 9.5)	28	753.72	3.7 (2.5 - 5.4)

All-cause death	515	1683.76	30.6 (28.0 - 33.3)	270	1841.51	14.7 (13.0 - 16.5)	104	902.34	11.5 (9.4 – 14)

*Primary outcome: hospitalisation for heart failure (hHF) or CV death.

Supplementary Table S5. Full Table for the Primary Outcome with All HF Patients.

Cohort	Unadjusted hazard ratio (95% CI)	p value	Adjusted hazard ratio* (95% CI)	p value
**In-patients vs. Out-patients**	2.39 (2.12 – 2.70)	< 0.001	1.62 (1.38 – 1.89)	< 0.001
**HF subgroups**				
HFrEF	*reference*		*reference*	
HFmrEF	0.48 (0.39, 0.58)	<0.001	0.54 (0.44, 0.65)	<0.001
HFpEF	0.29 (0.24, 0.34)	<0.001	0.33 (0.27, 0.40)	<0.001
HF with unknown EF	1.16 (1.01, 1.32)	0.032	0.91 (0.78, 1.05)	0.184
**Age (in years)**	1.02 (1.01, 1.02)	<0.001	1.01 (1.00, 1.01)	0.034
**Male sex**	0.99 (0.88, 1.11)	0.808	0.80 (0.71, 0.91)	<0.001
**Scottish Index of Deprivation Quintile**				
1 (Most deprived)	*reference*		*reference*	
2	0.93 (0.75, 1.16)	0.536	0.92 (0.74, 1.14)	0.447
3	1.03 (0.85, 1.26)	0.739	0.90 (0.74, 1.11)	0.326
4	1.16 (0.98, 1.38)	0.083	1.05 (0.88, 1.26)	0.559
5 (Least deprived)	1.00 (0.82, 1.22)	0.991	0.97 (0.79, 1.18)	0.735
**eGFR (mL/min/1.73m^2)**	0.99 (0.99, 0.99)	<0.001	0.99 (0.99, 0.99)	<0.001
**Comorbidities**				
Atrial fibrillation	1.29 (1.15, 1.45)	<0.001	1.07 (0.92, 1.25)	0.373
Coronary artery disease	1.12 (1.00, 1.26)	0.059	0.92 (0.81, 1.04)	0.194
Chronic kidney disease	1.63 (1.43, 1.85)	<0.001	1.13 (0.96, 1.34)	0.145
Chronic obstructive pulmonary disease	0.95 (0.81, 1.11)	0.489	1.02 (0.87, 1.19)	0.853
Diabetes	1.16 (1.02, 1.31)	0.024	1.20 (1.05, 1.37)	0.007

**Recently prescribed medications†**				
ACE inhibitors/ARBs/ARNI	1.00 (0.89, 1.12)	0.996	0.96 (0.85, 1.09)	0.567
Beta blockers	1.06 (0.94, 1.19)	0.315	0.98 (0.85, 1.13)	0.796
MRAs	1.19 (0.97, 1.46)	0.100	1.21 (0.97, 1.51)	0.084
Loop diuretics	0.85 (0.76, 0.96)	0.007	0.96 (0.84, 1.10)	0.601
Aspirin	1.08 (0.94, 1.24)	0.255	1.11 (0.95, 1.29)	0.197
Statins	0.97 (0.84, 1.11)	0.632	0.95 (0.82, 1.11)	0.513
DOACs	1.14 (0.98, 1.32)	0.083	1.10 (0.91, 1.33)	0.335
Warfarin	0.91 (0.71, 1.16)	0.450	0.91 (0.70, 1.18)	0.468

* The hazard ratios were obtained from a multivariable Cox regression adjusted for: age at diagnosis of de novo HF, sex, deprivation quintile, baseline eGFR, baseline comorbidities (atrial fibrillation, coronary artery disease, CKD, COPD, diabetes), and recently prescribed medications (ACE inhibitors/ARBs/ARNI, beta blockers, MRAs, loop diuretics, aspirin, statins, DOACs, and warfarin).

† Recently prescribed medications: medications prescribed within 365 days prior to diagnosis of de novo HF.

HFrEF – heart failure with reduced ejection fraction; HFmrEF – heart failure with mildly-reduced ejection fraction; HFpEF – heart failure with preserved ejection fraction; eGFR – estimated glomerular filtration rate; ACE – angiotensin converting enzyme; ARB – angiotensin II receptor blocker; ARNI – angiotensin receptor neprilysin inhibitor; MRA – mineralocorticoid receptor antagonist; DOAC – direct oral anticoagulant

Supplementary Table S6. Relative risk of the primary outcome (CV death or hHF) in in-patients compared to out-patients in the 365 days following diagnosis of de novo HF after exclusion of 204 patients with elective hospitalisation as their first event.

	**CV death or any hHF**		**CV death or emergency hHF**	
**Cohort**	**Adjusted HR* (95% CI)**	**p value**	**Adjusted HR* (95% CI)**	**p value**
**All HF patients**	1.62 (1.39 – 1.90)	<0.001*	1.88 (1.58 – 2.24)	<0.001*
**HF subgroups†**				
HFrEF	1.27 (1.04 – 1.55)	0.020*	1.49 (1.18 – 1.88)	0.001*
HFmrEF	1.52 (1.03 – 2.23)	0.034*	1.52 (1.00 – 2.32)	0.049*
HFpEF	3.28 (2.37 – 4.55)	<0.001*	3.55 (2.51 – 5.01)	<0.001*

HFrEF – heart failure with reduced ejection fraction; HFmrEF – heart failure with mildly-reduced ejection fraction; HFpEF – heart failure with preserved ejection fraction

Supplementary Figure S1. Kaplan-Meier Plot of the Primary Outcome in In-patient HFrEF/HFmrEF patients vs. Out-patient HFrEF/HFmrEF and non-HF Comparators.

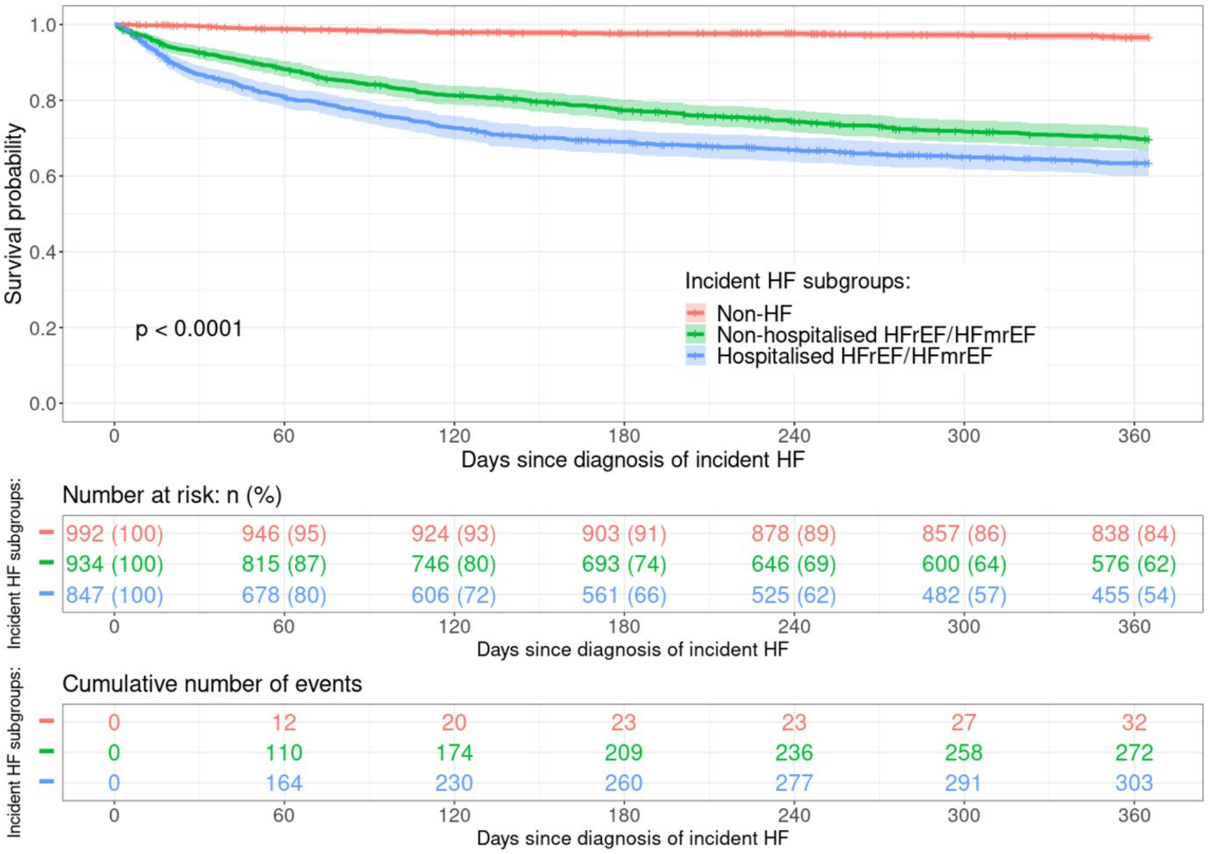

Supplementary Figure S2. Comparison Between In-patients and Out-patients on Secondary Outcomes in the 365 Days post Initial HF Diagnosis.

Secondary outcomes	Adjusted HR* (95% CI)	p
Hospitalisation for HF		
All HF	1.56 (1.31, 1.86)	<0.001*
HFrEF	1.16 (0.93, 1.44)	0.195
HFmrEF	1.48 (0.95, 2.30)	0.082
HFpEF	3.92 (2.63, 5.86)	<0.001*
CV death		
All HF	1.87 (1.45, 2.40)	<0.001*
HFrEF	1.90 (1.34, 2.70)	<0.001*
HFmrEF	1.89 (1.02, 3.51)	0.045*
HFpEF	2.05 (1.25, 3.37)	0.005*
Hospitalisation for MI		
All HF	1.84 (1.53, 2.21)	<0.001*
HFrEF	1.83 (1.40, 2.40)	<0.001*
HFmrEF	2.51 (1.79, 3.52)	<0.001*
HFpEF	1.55 (0.94, 2.56)	0.083
Hospitalisation for stroke		
All HF	1.50 (0.97, 2.32)	0.070
HFrEF	1.27 (0.70, 2.32)	0.437
HFmrEF	1.35 (0.46, 4.01)	0.585
HFpEF	1.54 (0.66, 3.59)	0.320
Worsening renal function		
All HF	1.33 (1.05, 1.70)	0.020*
HFrEF	1.32 (0.93, 1.88)	0.120
HFmrEF	2.76 (1.50, 5.09)	0.001*
HFpEF	1.01 (0.65, 1.58)	0.954
De novo CKD		
All HF	1.24 (0.91, 1.68)	0.182
HFrEF	1.32 (0.84, 2.07)	0.225
HFmrEF	0.99 (0.48, 2.04)	0.969
HFpEF	1.94 (1.07, 3.51)	0.029*
All-cause death		
All HF	1.65 (1.35, 2.01)	<0.001*
HFrEF	1.77 (1.31, 2.40)	<0.001*
HFmrEF	1.31 (0.78, 2.21)	0.307
HFpEF	1.83 (1.30, 2.59)	0.001*

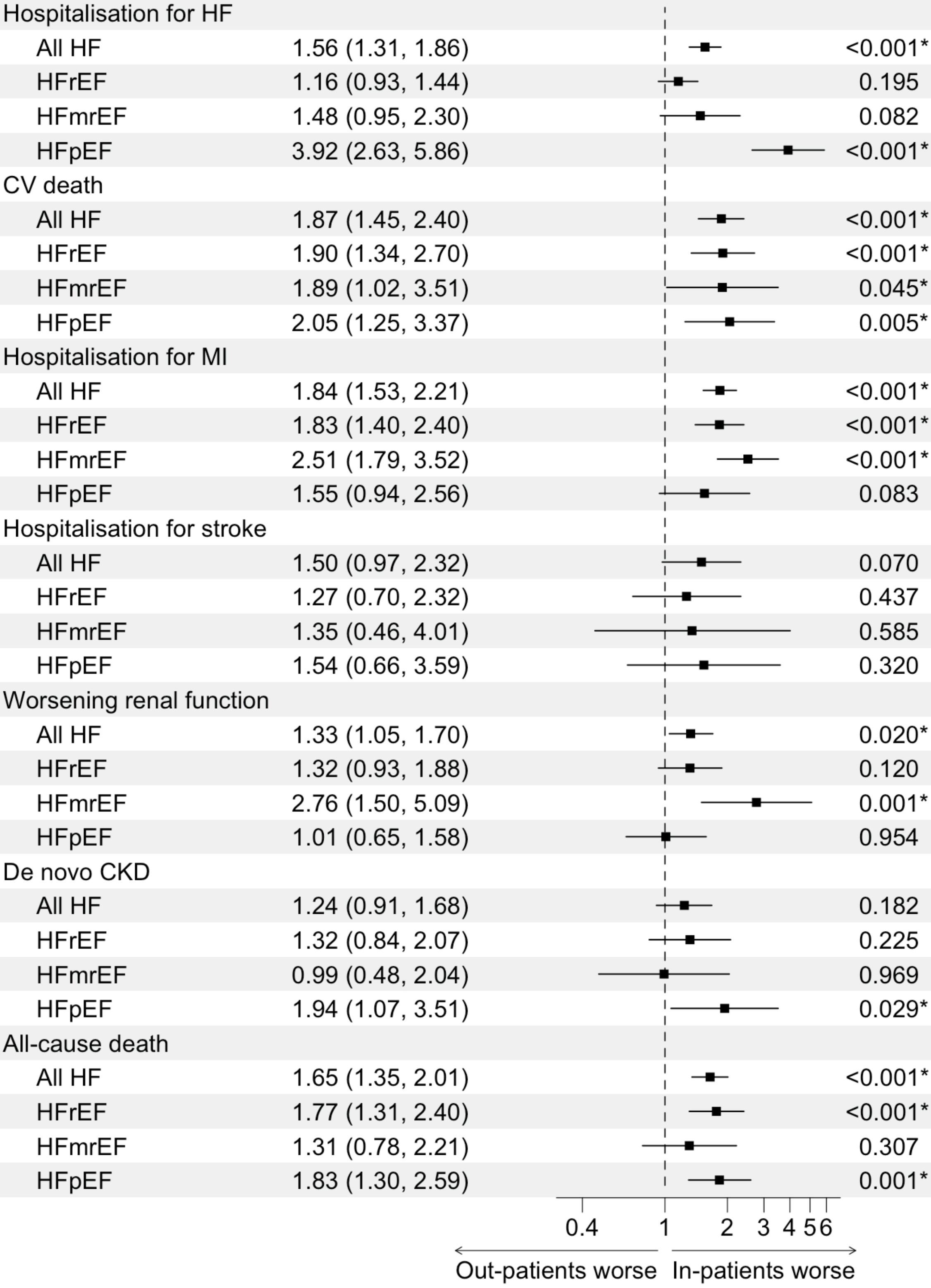

* The hazard ratios were obtained from a multivariable Cox regression adjusted for: age at diagnosis of de novo HF, sex, deprivation quintile, baseline eGFR, baseline comorbidities prior to diagnosis of de novo HF (atrial fibrillation, coronary artery disease, CKD, COPD, diabetes), and medications (ACE inhibitors/ARBs, beta blockers, MRAs, loop diuretics, aspirin, statins, DOACs, and warfarin) prescribed within 365 days prior to diagnosis of de novo HF.

(No stratified analysis was performed for HF patients with unknown EF as they were all diagnosed as in-patients [see Table 1]. This subgroup was included in the analysis of "All HF patients".)

Supplementary Figure S3. Comparison between 28-day or 60-day GDMT subgroups (among individuals with initial hospitalisation for HF with reduced ejection fraction only) on all-cause death and repeated hHF.

In-patients with HFrEF	2 or more GDMTs	0 or 1 GDMT	Adjusted HR* (95% CI)	p
28-day cohort (n = 478)	303	175		
Multivariable Cox			0.72 (0.55 - 0.94)	0.016*
IPTW			0.72 (0.54 - 0.95)	0.020*
PSM			0.72 (0.54 - 0.96)	0.024*
60-day cohort (n = 440)	323	117		
Multivariable Cox			0.74 (0.54 - 1.01)	0.059
IPTW			0.74 (0.53 - 1.05)	0.093
PSM			0.74 (0.52 - 1.05)	0.093

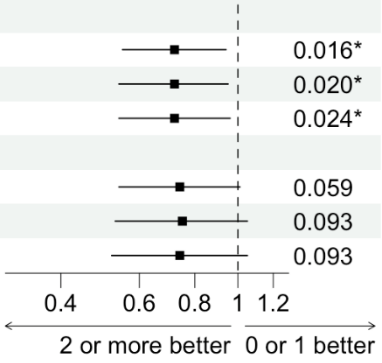

N-day cohort includes all in-patients with hHF-free survival for at least N days post discharge.

* The hazard ratios (HRs) were adjusted for: age at 28 days post discharge (from initial hHF), sex, deprivation quintile, baseline comorbidities prior to diagnosis of de novo HF (atrial fibrillation, coronary artery disease, CKD, COPD, and diabetes), and development of worsening renal function (WRF) during initial hHF.

IPTW: Inverse probability of treatment weighting. PSM: propensity score matching.
